# Contrasting Dispersal Histories Shape Distinct Evolutionary Trajectories Between Malesian and Pantropical *Talipariti* (Malvaceae)

**DOI:** 10.1002/ece3.74056

**Published:** 2026-07-30

**Authors:** Fernando Vélez‐Esperilla, Hiroshi Noda, Tadashi Kajita, Koji Takayama

**Affiliations:** ^1^ Department of Botany, Graduate School of Science Kyoto University Kyoto Japan; ^2^ Graduate School of Science Osaka Metropolitan University Katano Osaka Japan; ^3^ Iriomote Station, Tropical Biosphere Research Center University of the Ryukyus Okinawa Japan; ^4^ Makino Herbarium, Department of Biological Science, Graduate School of Science Tokyo Metropolitan University Tokyo Japan

**Keywords:** dispersal, herbariomics, Malesia, Malvaceae, palaeogeography, Sunda‐Sahul floristic exchange, *Talipariti*

## Abstract

The complex tectonic history of Malesia makes it a critical region for understanding the origins of tropical biodiversity, yet the drivers of plant diversification and range expansion across this region remain poorly resolved. *Talipariti* (Malvaceae) provides a unique system to test those drivers across Malesia since the Miocene. We aim to test whether species distributions were shaped primarily by stochastic long‐distance dispersal (LDD) or by stepping‐stone dispersal associated with tectonic collisions, by comparing two *Talipariti* lineages with contrasting geographic ranges: a pantropical sea hibiscus group and an endemic Papuan group. We constructed a phylogenomic framework using genome‐wide nuclear loci and plastome data, estimated divergence times, and applied quantitative biogeographical modelling to compare alternative dispersal scenarios, explicitly testing the contribution of founder‐event speciation and incorporating time‐stratified palaeogeographical information. *Talipariti* originated within Malesia ca. 20 Ma. A vicariance event along Lydekker's Line separated the Papuan group to the east from the remaining lineages to the west at ca. 8 Ma, and subsequently, the sea hibiscus group underwent rapid radiation from ca. 1.4 Ma. Biogeographical models reveal a pronounced dichotomy: the sea hibiscus group is best explained by unconstrained models incorporating founder‐event speciation, whereas the Papuan group is best supported by time‐stratified distance‐dependent models without a significant contribution of founder events. Pantropical distributions in the sea hibiscus group arose primarily through effective LDD events, whereas the Papuan group distribution expanded via stepping‐stone dispersal associated with Miocene Sunda‐Sahul island connectivity. These findings demonstrate that the evolutionary fate of plant lineages is not determined by geological history alone, but by the interaction between geological history and lineage‐specific dispersal abilities, revealing Wallace's and Lydekker's Lines as permeable filters rather than absolute barriers. Through biogeographical model comparison, this study provides a quantitative framework for understanding how geological history and dispersal processes jointly shape plant diversification across Malesia.

## Graphical abstract

*Talipariti* (Malvaceae) comprises two lineages with contrasting geographic ranges across Malesia: a pantropical sea hibiscus group and a Papuan group restricted to New Guinea and adjacent islands. Using phylogenomics, divergence‐time estimation, and quantitative biogeographical modelling applied to herbarium‐derived genomic data, we demonstrate that the pantropical distribution of the sea hibiscus group arose through rare long‐distance dispersal events, whereas the Papuan group expanded via stepping‐stone dispersal constrained by Miocene Sunda–Sahul island connectivity. These contrasting histories reveal that Wallace's and Lydekker's Lines function as permeable filters rather than absolute barriers, determined by lineage‐specific dispersal capacity.

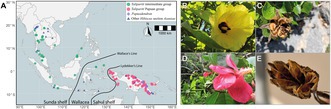

## 1 Introduction

The Malesian region (*sensu* Takhtajan 1986) is one of the world's most important natural laboratories for studying plant biogeography. Spanning Southeast Asia and Oceania, it encompasses a complex assemblage of continental shelves, island arcs and oceanic islands, and harbours exceptional species richness and endemism. Throughout the Cenozoic, repeated tectonic collisions, island emergence, and sea‐level fluctuations have continuously reshaped this region, generating some of the sharpest biogeographical discontinuities on Earth.

**FIGURE 1:**
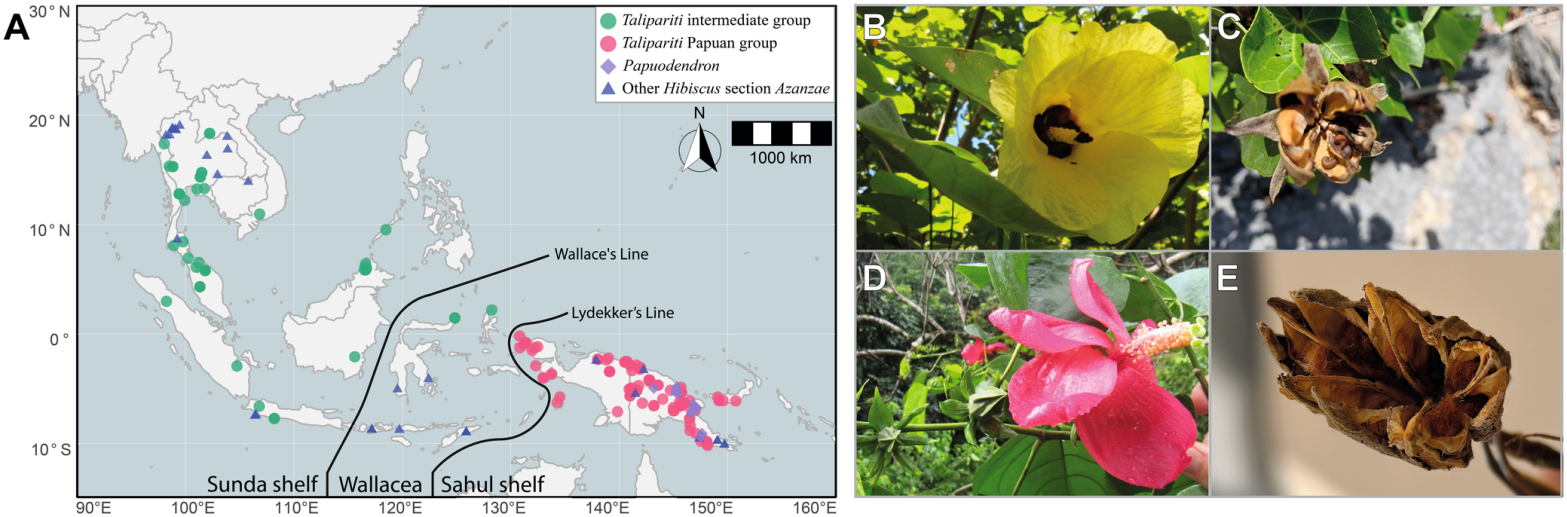
Distribution and morphology of Malesian *Talipariti* and allied species of *Hibiscus* section *Azanzae*. (A) Map of the Malesian region showing its three principal biogeographical areas (Sunda Shelf, Wallacea and Sahul Shelf), their boundaries (Wallace's Line and Lydekker's Line), and the distribution of sampled taxa based on georeferenced herbarium specimen data. The sea hibiscus group is omitted from the map for clarity given its broad distribution. (B–E) Representative images of *Talipariti*: (B, C) *T. tiliaceum* (sea hibiscus group); (D–E) Papuan group, (D) *T. archboldianum*, (E) *T. dalbertisii*. (B, D) Flowers; (C, E) dehisced 10‐celled capsules with seeds. Photo credits: B, C, E: Fernando Vélez‐Esperilla; D: Kelsey Wheeler (Waimea Valley, Hawaiʻi).

Species distributions in Malesia are primarily partitioned across the Sunda Shelf (comprising Mainland Southeast Asia, Borneo, Sumatra and Java), Wallacea (including Sulawesi, the Moluccas and the Lesser Sunda Islands), and the Sahul Shelf (encompassing New Guinea, its surrounding islands and Australia). Wallace's Line is one of the first and most widely recognised biogeographical barriers identified, separating taxa between Sunda and Wallacea (Wallace 1860). Floristic disjunctions, however, are more pronounced between Wallacea and Sahul, along Lydekker's Line (Lydekker 1896) (Figure 1A).

The tectonic convergence of the Sunda and Sahul shelves occurred approximately 25 Ma, following the northward drift of Sahul from Antarctica at ~45 Ma. At the Oligocene‐Miocene boundary, the Sahul continental shelf collided with the Wallacea archipelago and Sunda Shelf (Hall 2001). Because no persistent land bridge connected these continental blocks, biotic exchanges across Malesia have largely depended on overwater dispersal. Nevertheless, from ca. 12 Ma onward, the progressive emergence of island arcs established stepping‐stone corridors linking Sunda, Wallacea and Sahul (Hall 2001; Crayn et al. 2015). These palaeogeographical configurations are hypothesised to have facilitated the Sunda–Sahul floristic exchange, a pattern corroborated by molecular phylogenetic studies documenting significant lineage disjunctions dated to this period (Crayn et al. 2015; Joyce et al. 2021; Holzmeyer et al. 2023).

Despite decades of research, a central question remains unresolved: to what extent have Malesian plant distributions been shaped by rare, stochastic long‐distance dispersal events, versus dispersal constrained by short‐distance island connectivity associated with tectonic reorganisation? Resolving this question is critical for understanding how geological history and intrinsic biological traits interact to shape tropical plant diversification (Crayn et al. 2015; Holzmeyer et al. 2023). Yet, robust lineage‐level tests integrating phylogeny, timing, and palaeogeography remain surprisingly scarce (Appelhans et al. 2018; Shee et al. 2020).

*Hibiscus* sensu lato (Malvaceae, Malvoideae, tribe Hibisceae) provides a powerful system for addressing these questions owing to its wide geographic distribution, morphological diversity, and contrasting dispersal strategies. *Talipariti* Fryxell represents one of the lineages nested within *Hibiscus* s.l. and broadly corresponds to a subset of 23 species previously assigned to *Hibiscus* section *Azanzae* (Fryxell 2001; Bovini 2010). However, ten *Hibiscus* species not assigned to *Talipariti* remain within *Hibiscus* section *Azanzae* (Borssum Waalkes 1966; Dalavi et al. 2025); and, species of genus *Papuodendron* appear nested within *Talipariti* in Malvaceae‐wide phylogenetic analyses (Pfeil and Crisp 2005; Baker et al. 2022) (Table 1; Figure 1).

**TABLE 1:** List of *Talipariti* species classified into three subgroups, and an additional group of related species of *Hibiscus* section *Azanzae*, including categorical distribution range information and sampling status.

	Species	Distribution areas	Sampling
Sea hibiscus group	*Talipariti elatum* (Sw.) Fryxell	N	✓
	*Talipariti glabrum* (Matsum. ex Nakai) Fryxell	P	✓
	*Talipariti hamabo* (Siebold & Zucc.) Fryxell	P	✓
	*Talipariti hastatum* (L.f.) Fryxell	P	✓
	*Talipariti pernambucense* (Arruda) Bovini	N	✓
	*Talipariti potteri* (O.Deg. & Greenwell) Fryxell	P	✓
	*Talipariti simile* (Blume) Fryxell	A I	✓
	*Talipariti tiliaceum* (L.) Fryxell	PAIWG	✓
	*Talipariti tortuosum* (Roxb.) Fryxell	A	—
Intermediate group	*Talipariti borneense* (Airy Shaw) Fryxell	W	✓
	*Talipariti celebicum* (Koord.) Fryxell	W	✕
	*Talipariti crestaense* (Borss.Waalk.) Fryxell	W	✕
	*Talipariti macrophyllum* (Roxb. ex Hornem.) Fryxell	A I	✓
	*Talipariti pseudotiliaceum* (Borss.Waalk.) Fryxell	W	✓
Papuan group	*Talipariti archboldianum* (Borss.Waalk.) Fryxell	G	✓
	*Talipariti aruense* (Hatus. ex Borss.Waalk.) Fryxell	G	✓
	*Talipariti bowersiae* Fryxell	G	—
	*Talipariti dalbertisii* (F.Muell.) Fryxell	G	✓
	*Talipariti ellipticifolium* (Borss.Waalk.) Fryxell	G	✓
	*Talipariti leeuwenii* (Borss.Waalk.) Fryxell	G	✕
	*Talipariti pleijtei* (Borss.Waalk.) Fryxell	G	✓
	*Talipariti schlechteri* (Lauterb.) Fryxell	G	✓
	*Talipariti sepikense* (Borss.Waalk.) Fryxell	G	✓
*Hibiscus* section *Azanzae* non‐*Talipariti*	*Papuodendron hooglandianum* (Kosterm.) Borss.Waalk.	G	✓
	*Papuodendron lepidotum* C.T.White	G	✓
	*Hibiscus carrii* Borss.Waalk.	G	—
	*Hibiscus decaspermus* Koord. & Valeton	I W	✓
	*Hibiscus floccosus* Mast.	I	✓
	*Hibiscus glanduliferus* Craib	I	✓
	*Hibiscus mesnyi* Laness.	I	✕
	*Hibiscus pulvinulifer* Borss.Waalk.	G	✓
	*Hibiscus sciadiolepidus* (Hochr.) Borss.Waalk.	G	✓
	*Hibiscus teijsmannii* Borss.Waalk.	W	—

*Note:* Distribution range labels correspond to the following study areas: Pacific Islands (P), Neotropical (N), Afrotropical (A), Indochina and Sunda Shelf (I), Wallacea (W), and New Guinea and Sahul Islands (G). Sampling effort as follows: (✓) Sampled and successfully sequenced, (✕) sampled but not successfully sequenced and (—) sampling unavailable due to specimen conservation. Species list compiled from Borssum Waalkes (1966), Fryxell (2001), Bovini (2010), Dalavi et al. (2025).

Within *Talipariti*, two major lineages with contrasting geographical distributions and morphologies can be distinguished. The sea hibiscus group is characterised by 8–12 epicalyx segments, yellow corollas, and minute hairy to subglabrous seeds (Figure 1B), whereas the Papuan group has 5–8 epicalyx segments, reddish corollas and long hairy seeds (Figure 1C). Intermediate taxa also occur, combining features of both groups: some, such as *T. macrophyllum* , share the floral morphology of the sea hibiscus group but bear long hairy seeds, while others, such as *T. borneense*, resemble the Papuan group but have 8–10 epicalyx segments and white corollas.

The sea hibiscus group is derived from the widespread *T. tiliaceum* , and has a pantropical distribution that likely originated through oceanic dispersal. In *T. tiliaceum* , seed dispersal is facilitated by an internal air chamber which allows seed buoyancy and overwater transport, whereas derived species such as *T. glabrum* lack this structure and consequently show a reduction in dispersal ability (Kudoh et al. 2006, 2013). This lineage has served as a model system for studying long‐distance dispersal (LDD) and subsequent diversification, particularly in island environments (Takayama et al. 2005, 2006, 2008; Yamazaki et al. 2023). In contrast, the Papuan group comprises species largely restricted to the Sahul Shelf, including New Guinea and adjacent islands (Borssum Waalkes 1966; Fryxell 2001; Figure 1A). Seed anatomy and buoyancy have not been investigated in this group, and no evidence for oceanic dispersal adaptation is currently available, making the mechanisms underlying its geographical range unclear.

The restricted distribution of the Papuan group represents a biogeographical paradox. Despite its confinement to Sahul, its presence necessarily implies a historical crossing from Sunda or Wallacea. Two alternative scenarios may explain this pattern: colonisation via infrequent but effective, stochastic long‐distance dispersal events followed by loss of dispersal ability, or stepwise range expansion facilitated by short‐distance island connectivity during Neogene tectonic reorganisation of the Sunda–Sahul region (i.e., the Sunda‐Sahul floristic exchange; Crayn et al. 2015; Joyce et al. 2021). Discriminating between these hypotheses requires an explicit, quantitative framework integrating phylogenetic relationships, divergence times, species distributions, and palaeogeographical reconstructions.

Addressing these questions is challenging because members of the Papuan group are often rare, geographically restricted and difficult to sample. Many species occur in remote, topographically complex regions, limiting the availability of fresh material suitable for genomic analyses. Recent advances in herbarium genomics (‘herbariomics’), particularly target enrichment approaches, now allow the recovery of phylogenomic‐scale data from highly degraded DNA, enabling the inclusion of historical and rare specimens in evolutionary analyses (Brewer et al. 2019; Shee et al. 2020).

A further challenge arises from the complex evolutionary history of Malvaceae, where rapid radiations, incomplete lineage sorting (ILS), genome duplication, and recombination events cause widespread gene‐tree discordance (Colli‐Silva et al. 2025; Yang et al. 2025). In *Talipariti* and related lineages, high chromosome numbers and evidence of ancient polyploidy further complicate phylogenetic inference (Skovsted 1935; Marinho et al. 2014; Luna et al. 2025). Robust reconstruction of species relationships therefore requires phylogenomic approaches based on hundreds of loci and explicit consideration of gene‐tree conflict (Johnson et al. 2019; Baker et al. 2022; Hernández‐Gutiérrez et al. 2022; Colli‐Silva et al. 2025; Luna et al. 2025).

Here, we generate the first comprehensive, time‐calibrated phylogeny of *Talipariti* as a whole using nuclear and plastid genomic data derived from either silica gel‐dried leaf tissue or herbarium specimens. Building on this framework, we reconstruct the historical biogeography of these lineages across Malesia using quantitative, time‐stratified biogeographical models. Specifically, we test whether the colonisation of Sahul was driven primarily by stochastic long‐distance dispersal or by stepping‐stone dispersal associated with Neogene tectonic reorganisation. We further assess whether these mechanisms differ between the pantropical sea hibiscus group and the geographically restricted Papuan group.

## 2 Materials and Methods

### 2.1 Sampling and DNA Extraction

Samples were obtained either from living individuals (silica gel‐preserved) or from herbarium specimens. Herbarium specimens were examined and sampled in situ when permitted by specimen conservation status, from the collections housed at the Naturalis Biodiversity Center (Netherlands; L), the Royal Botanic Gardens, Kew (UK; K) and the Kyoto University Museum (Japan; KYO). Our sampling represents 21 of the 23 described species of *Talipariti* (Fryxell 2001; Bovini 2010) as well as closely related taxa, including the two species of *Papuodendron* and six of the eight remaining species within *Hibiscus* section *Azanzae* (Borssum Waalkes 1966) (Table 1). Sampling was conducted during visits to each herbarium, prioritising specimens for which sampling had minimal impact on specimen preservation. Silica gel‐preserved samples, which were only available for the sea hibiscus group, were sourced from our previous studies (Takayama et al. 2005, 2006, 2008; Yamazaki et al. 2023), with additional specimens included in this work. Details of samples included in the final dataset are provided in Table S1.1 and S1.2.

Leaves were pulverised using a TissueLyser (QIAGEN, Germany). Different DNA extraction protocols were applied depending on the sample origin. For silica gel‐preserved samples, we used the Plant Genomic DNA Extraction Mini Column (Favorgen Biotech, Taiwan). For herbarium samples, we applied a modified CTAB protocol (Doyle and Doyle 1987) following the protocol of Fairlie & Pokorny (see Supporting Information in Larridon et al. 2020), which includes an increased concentration of β‐mercaptoethanol and extended incubation times (Larridon et al. 2020; Shee et al. 2020). In total, DNA was extracted from more than 100 herbarium specimens, including material collected as early as 1892.

### 2.2 Gene Library Preparation, Enrichment and Next‐Generation Sequencing

Library preparation protocols were selected according to the degree of DNA fragmentation. We used xGen DNA Library Preparation Kits (IDT, Iowa, USA): the EZ kit (enzymatic fragmentation) for silica gel‐preserved samples and the MC kit (without additional shearing) for herbarium samples, in which DNA was already highly fragmented, using 12 PCR cycles in both cases. For each sample, libraries were split into two fractions: one was amplified and sequenced directly to recover high‐copy plastid DNA (genome skimming), and the other was enriched with Angiosperms353 baits, using 14 PCR cycles for enrichment (Johnson et al. 2019; myBaits Expert Angiosperms‐353, Arbor Biosciences, USA) to target single‐copy nuclear loci (target‐enriched sequences).

DNA sequencing was performed using 150‐bp paired‐end reads on the DNBSEQ‐G400 platform (BGI, China). Quality control was conducted at multiple stages, including DNA concentration using Invitrogen Qubit Fluorometer 2.0 (Thermo Fisher Scientific, USA) and fragment‐size assessment using MultiNA (Shimadzu, Japan), following target capture protocol recommendations (Hale et al. 2020). All demultiplexed reads were trimmed and filtered using fastp v0.23.4 (Chen 2023).

### 2.3 Phylogenomics Analyses

For phylogenomic analyses, we combined newly generated sequences with publicly available data. For the plastome dataset, we incorporated 63 complete plastome sequences downloaded from NCBI (www.ncbi.nlm.nih.gov) and 4 sequences from our previous study (Yamazaki et al. 2023). For the target‐enriched dataset, we downloaded raw Angiosperms353 FASTQ files from the PAFTOL project (Baker et al. 2022), additional raw reads from the European Nucleotide Archive (www.ebi.ac.uk/ena), and the transcriptome of *Sabdariffa cannabina* from OneKP (Matasci et al. 2014). Details of downloaded genomic data are provided in Table S1.3.

### 2.4 Plastome Phylogeny

Shotgun sequencing reads were assembled by mapping against the reference plastome of *Talipariti hamabo* (LC554222; Yamazaki et al. 2023) using geneious prime 2020.1.2 (Biomatters, New Zealand). Custom sensitivity settings were applied (maximum gap size: 100; maximum ambiguity: 5). 38 newly generated assemblies with greater than 95% reference coverage were retained, which combined with the downloaded data yielded a total of 143 consensus sequences for downstream analyses.

Consensus sequences were initially aligned with mafft 7.520 (Katoh and Standley 2013) with the ‘auto’ strategy, and the alignment was annotated in geneious prime by transferring annotations from the reference plastome (70% similarity threshold), with manual adjustment of coding regions to preserve reading frames. Unassembled positions were coded as ‘N’. Newly annotated plastomes were extracted from the alignment and submitted to GenBank (BioProject accession number: PRJNA1496457).

For phylogenetic analyses, in all plastomes the inverted repeat A (IRA) regions were removed, and the small single‐copy (SSC) region was reverse‐complemented when necessary to ensure consistent gene orientation. Alignments were generated using mafft 7.520 (Katoh and Standley 2013) with the ‘auto’ strategy and filtered with trimal (‐gt 0.1 ‐cons 35) (Capella‐Gutiérrez et al. 2009). After manual inspection and removal of ambiguous regions, parsimony‐informative sites were extracted using amas 1.0 (Borowiec 2016) and maximum‐likelihood (ML) inference with 1000 ultrafast bootstrap replicates was performed using iq‐tree 2.2.6 (Nguyen et al. 2015). A separate CDS dataset comprising 75 concatenated plastid coding regions was extracted and analysed using the same pipeline.

### 2.5 Hyb‐Seq Phylogeny

Target‐enriched reads were assembled using hybpiper 2.1.6 (Johnson et al. 2016) with the mega353.fasta target file (McLay et al. 2021) and a minimum coverage of 3. Paralog detection and recovery statistics were evaluated within hybpiper. To reduce alignment noise, we followed the filtering strategy of Pokorny et al. (2024). Samples with low locus recovery were excluded using the ‘max_overlap.R’ script (Shee et al. 2020). As multiple accessions per species were available for the sea hibiscus group, up to four representative accessions per taxon were retained based on their coverage scores as ranked by ‘max_overlap.R’, ensuring both adequate phylogenetic representation and balanced sampling across lineages.

Supercontig alignments were generated for 267 single‐copy loci with hybpiper. Alignments were conducted with mafft 7.520 (Katoh and Standley 2013), exploratory gene trees inferred with fasttree 2.1.11 (Price et al. 2010) and extreme outliers removed using treeshrink 1.3.9 (Mai and Mirarab 2018). Alignment statistics were calculated using amas 1.0 (Borowiec 2016), after which alignments were realigned with mafft and quality filtered with trimal 1.4.1 (−gt 0.1 ‐cons 35) (Capella‐Gutiérrez et al. 2009).

Phylogenetic inference was conducted using both concatenation and coalescent‐based approaches. For concatenation, loci were combined into a supermatrix (743,810 bp; 125 samples) using amas, and maximum‐likelihood trees were inferred with raxml‐ng (Kozlov et al. 2019) using the best‐fit substitution model determined by modeltest‐ng (Darriba et al. 2020). For coalescent analyses, individual gene trees were inferred with iq‐tree 2.2.6 (Nguyen et al. 2015), with branch support assessed from 1000 bootstrap replicates. Gene trees were then processed to reduce noise: outlier branches were removed with treeshrink v1.2.1 (Mai and Mirarab 2018), and branches with very low support (< 20%) were collapsed using newick utilities 1.6 (Junier and Zdobnov 2010). The processed gene trees were used for species tree inference with astral‐pro2 1.15.1.3 (Zhang et al. 2020; Zhang and Mirarab 2022).

Gene‐tree discordance was assessed by comparing gene trees to the ASTRAL species tree using Phytop 0.3.2 (Shang et al. 2025). Tanglegrams were generated to visualise incongruence among nuclear and plastome trees with the ‘cophylo’ function in the r package ‘phytools’ (Revell 2024).

### 2.6 Time Calibration

To avoid heterogeneity in evolutionary rates across nuclear loci (Hernández‐Gutiérrez et al. 2022), divergence‐time estimation was conservatively based on our highest‐quality plastome alignment, specifically the CDS. Sampling comprised one accession per species, with the following exceptions: *T. tiliaceum* , for which three accessions were included to represent Atlantic, Indic and Pacific lineages (Yamazaki et al. 2023), and *T. archboldianum*, for which two accessions were included due to its polyphyletic placement in the phylogeny.

We selected two fossil calibrations showing clear morphological affinity with extant Malvoideae taxa. The first calibration was the widely used leaf fossil *Malvaciphyllum macondicus* M. Carvalho, dated to 58–60 Ma and assigned to the stem lineage of Eumalvoideae, including *Howittia* and *Lagunaria* (Carvalho et al. 2011; Hernández‐Gutiérrez and Magallón 2019; Cvetković et al. 2021). The second calibration was the recently described infructescence fossil with schizocarps, *Uiher karuen* Siegert, Gandolfo, et Wilf dated at 52.0–52.44 Ma and assigned to the tribe Malveae within Malvoideae (Siegert et al. 2024).

Divergence‐time analyses were performed using ‘mcmctree’ (Álvarez‐Carretero et al. 2022; García‐Mir 2025) as implemented in paml 4.10.9 (Yang 1997, 2007). A soft maximum bound of 71.67 Ma was applied to the root age based on secondary calibration estimates for the Malvatheca clade (Bombacoideae and Malvoideae subfamilies) reported in the literature (Cvetković et al. 2021). Posterior divergence times were estimated using six independent MCMC chains, each run for 200,000 iterations following a burn‐in period of 2000 cycles. Convergence was assessed by examining the potential scale reduction factor (Rhat) and effective sample size (ESS) for all parameters and node ages.

### 2.7 Biogeographical Inference

Ancestral range estimation was conducted using the R package ‘BioGeoBEARS’ 0.2.1 (Matzke 2013a), based on our time‐calibrated phylogeny inferred from the CDS data and current species distributions compiled from herbarium specimens, supplemented by literature and online databases (Table S2.1). Six biogeographic regions were defined to represent the distributional ranges of *Talipariti* species: Pacific Islands (P), Neotropical (N), Afrotropical (A), Indochina and Sunda Shelf (I), Wallacea (W) and New Guinea and Sahul Islands (G).

To account for dispersal heterogeneity and reduce potential biases associated with widespread pantropical taxa, we partitioned the data into three subsets: (i) the full dataset (including all OTUs of *Talipariti, H*. *floccosus* and the genus *Kydia* as an outgroup); (ii) the sea hibiscus group dataset; and (iii) the Papuan group dataset. This partitioning strategy allowed us to assess model fit independently for each lineage.

To test the influence of geological and palaeogeographical history on species distributions, we compared three dispersal constrained scenarios across all datasets: M0, a null model assuming equal dispersal rates among all regions; M1, a palaeogeography‐informed model that treats Wallacea as a strict biogeographic barrier; and M2, a distance‐dependent model in which dispersal probability reflects paleogeographic proximity consistent with a stepping‐stone dynamic that varies through time. For M2, time‐stratified dispersal multiplier matrices were constructed to reflect major changes in island connectivity based on paleogeographic reconstructions of Malesia, including events such as the Sula Spur collision and the narrowing of the Makassar Strait (Hall 2001, 2013; Toussaint et al. 2014). Four temporal slices were defined: Oligocene (34–23 Ma), Early to Middle Miocene (23–10 Ma), Late Miocene to Pliocene (10–2.6 Ma) and Quaternary (2.6–0 Ma). Detailed information on OTUs, geographic distributions and dispersal matrices is provided in Table S2.1 and S2.2.

For each dispersal scenario (M0, M1, M2), we fitted six models implemented in BioGeoBEARS: DEC (Ree and Smith 2008); DIVALIKE, an approximation to DIVA (Ronquist 1997); and BAYAREALIKE, an approximation to Bay Area (Landis et al. 2013). All models were tested with and without the founder‐event speciation parameter (j). Model performance was compared using the Akaike Information Criterion (AIC). In addition, likelihood‐ratio tests (LRTs) were used to assess the statistical significance of the j parameter by comparing nested models (e.g., DEC vs. DEC + J).

## 3 Results

### 3.1 First Phylogenies of *Talipariti* Including Malesian Species

In the filtered Hyb‐Seq dataset, 50.3% of reads were mapped to the target genes. Gene capture was highly comprehensive across Malvaceae taxa, with only one of the 353 target loci completely absent from all samples. A median of 242 loci per sample was recovered with at least 50% of the target length assembled (Figure S1), and 267 loci remained as single‐copy loci for our study system (Figure S1). The final whole‐plastome alignment comprised 176,859 bp, including 16,722 bp parsimony‐informative sites. From this alignment, we extracted 75 concatenated CDS regions (65,848 bp: 5413 parsimony‐informative sites). Herbarium‐derived samples showed substantial variability in DNA quality (concentration, fragment length and dimer formation) throughout library preparation, resulting in a high attrition rate. Overall, 28.5% of herbarium samples were retained after sequencing and bioinformatic filtering. This variability showed no consistent association with taxon, specimen age, or herbarium provenance. The oldest specimen successfully sequenced in this study was collected nearly 120 years ago (*T. hastatum*, L.2371363).

Our species tree provides strong support for the monophyly of *Talipariti*, but only when *Papuadendron, H. sciadiolepidus*, *H. pulvinulifer* and *H. glanduliferus* are included within the genus (coalescent nuclear genes: posterior probability (PP) = 0.81; concatenated nuclear genes bootstrap (BS) = 100%; plastome concatenated CDS bootstrap (pBS) = 100%). A single exception was observed: *Talipariti borneense* (the intermediate group species resembling the Papuan group) was placed outside this clade, in a distinct Hibisceae lineage (*Trionum* clade), grouping with *Pavonia urens* in nuclear trees and *Hibiscus mutabilis* in plastome analyses.

**FIGURE 2:**
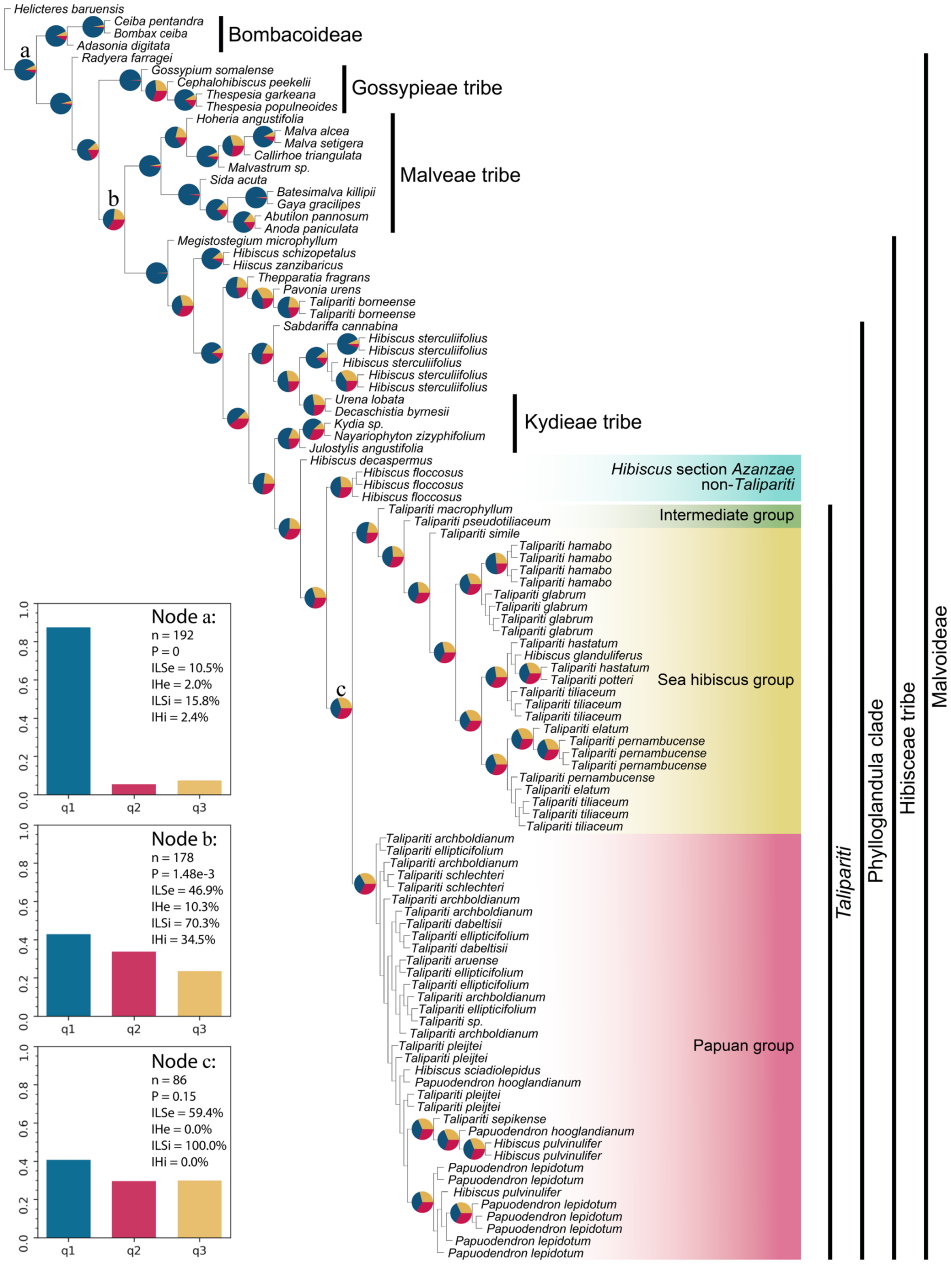
Coalescence‐based nuclear phylogeny of Malvaceae inferred from 267 single‐copy nuclear loci obtained from Angiosperms353 target‐enrichment data. Pie charts illustrate gene tree topologies: (q1, blue) topologies concordant with the species tree, and (q2, red and q3, yellow) conflicting alternative topologies. Three focal nodes were selected to examine gene‐tree conflict in detail (bar charts): (a) Malvoideae/Bombacoideae, (b) Hibisceae/Malveae and (c) Sea hibiscus/Papuan groups. Bar chart statistics: *N* is the number of gene trees informative for that node; *p* is the *p*‐value of the χ^2^ test assessing symmetry between topologies q2 and q3; ILS‐i and IH‐i denote the calculated indices for Incomplete Lineage Sorting and Introgression/Hybridization, respectively; and ILS‐e and IH‐e represent the proportion of topological incongruence explained by these processes. Pie charts are omitted on branches with a posterior probability < 0.7 in the ASTRAL species tree.

*Talipariti* is placed within the Phylloglandula clade and *Hibiscus* section *Azanzae*. Within Phylloglandula clade, taxa are divided into two groups: (i) a clade including *Sabdariffa* (synonym of *Hibiscus* section *Furcaria*; Barrett et al. 2025), the African species *Hibiscus sterculiifolius*, and the genera *Urena* and *Decaschistia*; and (ii) a clade comprising *Hibiscus* section *Azanzae* (including *Talipariti*), which is sister to the clade of genera *Kydia*, *Nayariophyton* and *Julostylis* (Figure 2). Within *Hibiscus* section *Azanzae*, a lineage comprising the west Malesian species *H. decaspermus* and *H. floccosus* is resolved as sister to *Talipariti* (PP = 0.88; BS = 100%; pBS = 100%), indicating an early divergence within the section prior to the diversification of *Talipariti*. Within *Talipariti*, three major lineages are distinguished: (i) the Papuan group, endemic to New Guinea and the surrounding islands; (ii) an intermediate group distributed on the Sunda Shelf and Wallacea, comprising *T. macrophyllum* , *T. pseudotiliaceum* and *H. glanduliferus*; and nested within this intermediate group, (iii) the sea hibiscus group, corresponding to the radiation of *T. tiliaceum* .

Within the Papuan group two principal clades were recovered with moderate support: one comprising large‐flowered species (*T. archboldianum, T. aruense*, *T. dalbertisii, T. ellipticifolium* and *T. schlechteri*), and another comprising small‐flowered species (*T. pleijtei* and *T. sepikense*), two *Hibiscus* section *Azanzae* non‐*Talipariti* species (*H. sciadiolepidus* and *H. pulvinulifer*), and the two species of *Papuodendron* (PP = 0.62; BS = 66%).

Across Malvaceae, inferred phylogenies recovered well‐supported clades at deep taxonomic levels (subfamily, tribe and genus), whereas support declined toward shallow nodes corresponding to species‐level relationships. Most deep nodes exhibited high concordance among gene trees and low ILS values. A notable exception is the relationship among Malveae, Hibisceae and Gossypieae, which showed elevated discordance (external ILS = 46.9%; internal ILS = 70.3%) (Figure 2B). Gene‐tree incongruence increased sharply toward shallow nodes resolving closely related species, a pattern particularly pronounced within the Phylloglandula clade and reaching its maximum within *Hibiscus* section *Azanzae* (Figure 2C). At these nodes, alternative topologies were recovered in nearly equal proportions, resulting in low astral local posterior probabilities (LPPs) and ILS values frequently approaching 100%.

The concatenated tree yielded a topology largely congruent with the coalescent species tree at deeper levels, but showed higher branch support values and recovered monophyly for some groups that appeared paraphyletic in the coalescent tree, for instance, the large‐flowered species lineage within the Papuan group (Figure S2).

**FIGURE 3:**
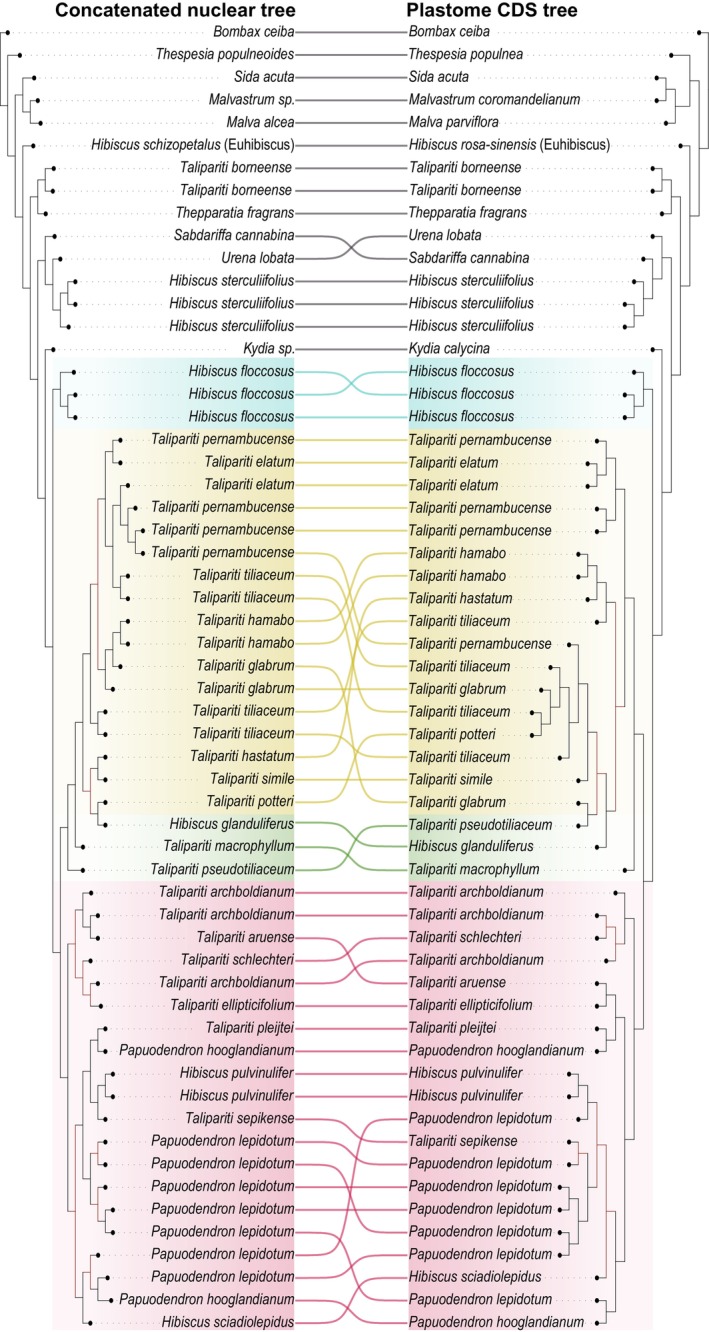
Tanglegram comparing cytonuclear discordance using nuclear concatenated and plastome CDS datasets. Red nodes indicate low support (BS < 70%). Colours represent *Hibiscus* section *Azanzae* groups: (teal) early diverged sister group of *Talipariti*, (yellow) the sea hibiscus group, (green) the intermediate group and (red) the Papuan group. For comparisons with the astral species tree, see Figures S2 and S3.

Plastome phylogeny was largely congruent with nuclear results, with one notable exception in tribal relationships: Malveae was recovered as sister to Gossypieae rather than to Hibisceae. Consistent with the nuclear analyses, *T. borneense* was excluded from the Phylloglandula clade and placed with maximum support within the *Trionum* clade (near *H. mutabilis* ) of the tribe Hibisceae. Excluding this species, the monophyly of the *Hibiscus* section *Azanzae* and its sister relationship to *Kydia* were strongly supported (BS = 100%), *Talipariti* sister group of west Malesian species (BS = 100%), the whole *Talipariti* monophyly (BS = 100%), and its major lineages (Papuan and sea hibiscus groups; both BS = 100%). The intermediate species *T. macrophyllum* and *H. glanduliferus* were also resolved as sisters to the sea hibiscus group. Different from nuclear genes estimations, *T. pseudotiliaceum* was nested within the *T. tiliaceum* clade (sea hibiscus group) and closely related to the Ogasawara endemic *T. glabrum* . Species‐level relationships within both the sea hibiscus and Papuan groups differ between plastome and nuclear phylogenies; most notably, *T. tiliaceum* is not monophyletic in both topologies (Figure 3, Figure S3).

### 3.2 Time Calibration

**FIGURE 4:**
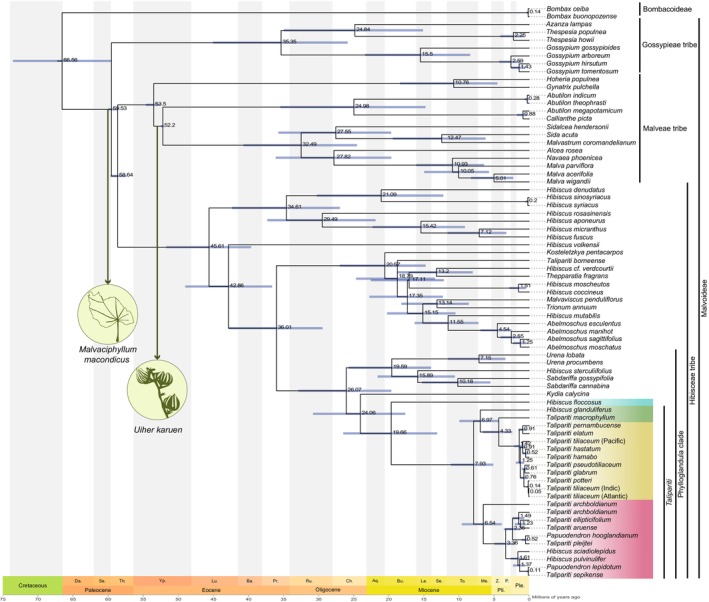
Time‐calibrated phylogeny of Malvoideae focusing on *Talipariti*, inferred from plastome CDS data (75 concatenated coding regions). Error bars on nodes indicate 95% highest posterior densities around the mean dates. Green arrows point to the nodes containing two fossil calibrations illustrated on circles: *Malvaciphyllum macondicus* and *Uiher karuen*. Colours on OTUs represent *Hibiscus* section *Azanzae* groups: (teal) early diverged sister group of *Talipariti*, (yellow) the sea hibiscus group, (green) the intermediate group and (red) the Papuan group.

MCMC diagnostics indicated good convergence, with Rhat values close to 1 and ESS values ranging from 1693 to 582,243 across all nodes (Table S3). Divergence‐time estimates placed the origin of Malvoideae at 66.5 Ma (95% HPD: 59.5–73.5 Ma), Hibisceae at 45.6 Ma (95% HPD: 39.6–51.7 Ma), and the Phylloglandula clade at 26 Ma (95% HPD: 19.6–32.9 Ma). Following its split from the former Kydieae genera at 24 Ma (95% HPD: 17.6–30.8 Ma), diversification within *Hibiscus* section *Azanzae* began at 19.6 Ma (95% HPD: 13.1–26.5 Ma), marked by the divergence of the lineage sister to *Talipariti* (*H. floccosus*) and the origin of *Talipariti*. The Papuan group subsequently diverged from the remaining *Talipariti* lineages (intermediate and sea hibiscus groups) at 7.9 Ma (95% HPD: 5.1–11.1 Ma) and underwent further diversification, with radiation in New Guinea beginning at 6.5 Ma (95% HPD: 3.9–9.6 Ma). The sea hibiscus group diverged from the intermediate lineage at 4.3 Ma (95% HPD: 2.4–6.6 Ma), with rapid radiation within the group initiating at ca. 1.4 Ma (95% HPD: 0.8–2.1 Ma) (Figure 4).

### 3.3 Biogeographical Inference

For both the complete dataset and the sea hibiscus group subset, the unconstrained dispersal scenario (M0) provided the best overall fit. Across all tested dispersal scenarios, DEC + J provided the best fit according to AIC, with likelihood‐ratio tests confirming a significant improvement in fit (*p* = 0.003). DEC + J also consistently outperformed the DIVALIKE and BAYAREALIKE models. Under the optimal DEC + J model, the estimated jump dispersal rate exceeded the anagenetic range expansion rate (*d* = 0.0058, *j* = 0.0155; Table S2.3).

**FIGURE 5:**
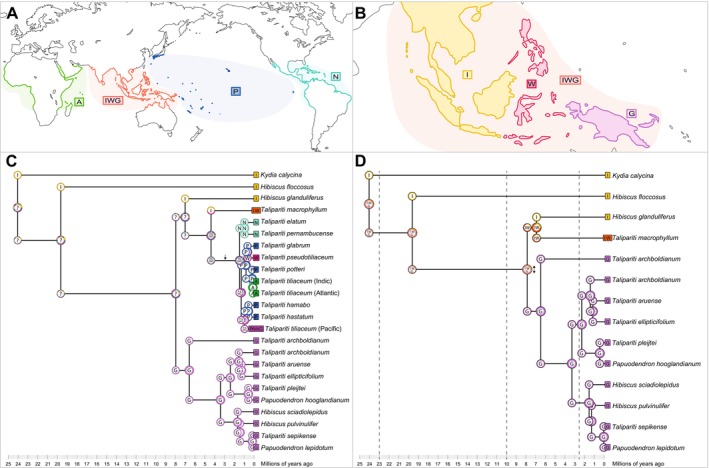
Ancestral range estimation of *Hibiscus* section *Azanzae* based on the time‐calibrated plastome CDS phylogeny. (A, B) Maps showing the biogeographical areas defined for the analysis: (A) Global view and (B) detailed view of the Malesian region. (C, D) Optimal ancestral range reconstructions. Pie charts at nodes represent the relative probability of ancestral ranges. (C) Analysis of the full dataset using the DEC + J model (scenario M0), the arrow marks the branch where pantropical expansion occurs. (D) Analysis of the Papuan group subset using the DEC model; dashed lines represent the changes in dispersal matrices in time‐stratified analysis (model M2, Table S2.2); double arrow marks the node from which vicariance event occurs. Area codes: A, Afrotropical; N, Neotropical; P, Pacific Islands; IWG, Malesia (composite); I, Indochina and Sunda Shelf; W, Wallacea; G, New Guinea and Sahul Shelf islands.

Ancestral range estimates for deep nodes were equivocal, including the common ancestor of *Hibiscus* section *Azanzae* and the outgroup *Kydia*, as well as the divergence of *H. floccosus* (*Talipariti* sister lineage) and the Papuan group. In contrast, reconstructions indicate a single evolutionary origin of LDD within *Talipariti*. Although ancestral states at deeper nodes remain uncertain, the probability of a widespread pantropical range increases progressively through successive divergences, reaching nearly 50% at the node separating *T. macrophyllum* from the sea hibiscus group (4.33 Ma). Following this split, the probability of a pantropical ancestral range increased to > 75% prior to the radiation of the sea hibiscus group at ca. 1.25 Ma. The inferred ancestral range of the sea hibiscus group encompassed Malesia, the Pacific, and the Neotropics, and subsequently contracted in the most derived species, with the widespread distribution retained only by *T. tiliaceum* (Figure 5A–C).

In the Papuan group dataset, the time‐stratified, distance‐dependent scenario (M2) showed the best performance. Under M2, DEC models again fit the data better than DIVALIKE and BAYAREALIKE, but LRTs did not support a significant contribution of founder‐event speciation (*p* > 0.99). Under this model, the estimated anagenetic range expansion (*d* = 0.0028) exceeded the inferred contribution of jump dispersal (Table S2.3). In contrast to results from the full dataset, DEC reconstruction under the M2 framework resolved Malesia as the ancestral area for the deep nodes of *Hibiscus* section *Azanzae* and for the divergence of *Talipariti* from its sister lineage (*H. floccosus*). This was followed by a vicariance event coinciding with Lydekker's Line, separating the intermediate lineage (*H. glanduliferus* and *T. macrophyllum* ) in Indochina/Sunda and Wallacea from the Papuan group in Sahul (Figure 5B,D).

## 4 Discussion

Biogeographical models that incorporate palaeogeographical connectivity have proven powerful for elucidating plant dispersal and diversification in the Malesian region (e.g., Holzmeyer et al. 2023). Although many plant lineages exhibit Sunda‐Sahul disjunctions, particularly since the Late Miocene (Crayn et al. 2015), relatively few studies have explicitly modelled plant dispersal and diversification in Malesia during this time using quantitative, time‐stratified frameworks (de Boer et al. 2015; Kolanowska et al. 2016; Appelhans et al. 2018). Moreover, to our knowledge, no previous studies have explicitly compared alternative dispersal mechanisms within a single Malesian plant clade, in a unified quantitative framework.

Our analyses demonstrate that dispersal dynamics within *Talipariti* differed markedly between lineages with contrasting dispersal syndromes. This contrast indicates that the influence of Sunda–Sahul geological reconfiguration was not uniform across lineages, but instead depended strongly on lineage‐specific dispersal abilities. Additionally, under both scenarios, Wallacea and its biogeographical limits (Wallace's and Lydekker's lines) do not act as strict barriers to species dispersal (model M1), but rather as dynamic and permeable filters, mediated by lineage‐specific dispersal abilities (models M0 and M2). This dynamic has been theorised for other plant lineages, including the genus *Begonia* (Thomas et al. 2012) and the genus *Didymocheton* (Holzmeyer et al. 2023).

In the following sections, we first discuss the biogeographical origin and phylogenetic structure of *Talipariti* and *Hibiscus* section *Azanzae*. We then examine how contrasting dispersal histories shaped evolutionary trajectories within *Talipariti*, before considering the temporal context of these patterns. Finally, we briefly address the implications of phylogenetic uncertainty for biogeographical inference and future directions in biogeographical model comparison.

### 4.1 Biogeographical Origin of *Talipariti* and *Hibiscus* Section *Azanzae*

The placement of *Hibiscus* section *Azanzae* close to the genera formerly assigned to Kydieae and distributed in South Asia is consistent with previous broad‐scale phylogenetic studies (Pfeil and Crisp 2005; Koopman and Baum 2008; Hernández‐Gutiérrez et al. 2022; Hanes et al. 2024; Colli‐Silva et al. 2025), as well as more focused analyses of *Hibiscus* and related lineages (Takayama et al. 2008; Yamazaki et al. 2023). Taken together, these results support a Sunda‐region origin of *Hibiscus* section *Azanzae*, followed by subsequent dispersal toward Sahul, and a Malesian origin of *Talipariti*. This scenario is independently corroborated by our biogeographical reconstructions for the Papuan group dataset. However, we note that ancestral range estimates at deeper nodes of the full dataset remain equivocal.

Our phylogenomic analyses further clarify the internal relationships within the major groups of *Talipariti*. The three morphological groups traditionally recognised (sea hibiscus, intermediate and Papuan) are recovered as well‐supported lineages that also show a striking geographical correspondence, with predominantly pantropical, Sunda and Sahul distributions, respectively. In contrast to the earlier classifications (Borssum Waalkes 1966; Fryxell 2001), New Guinean endemics previously assigned to either *Hibiscus*, *Talipariti*, or *Papuodendron* are nested within the Papuan group, indicating that the morphological and generic boundaries within this clade have not consistently reflected evolutionary relationships. The marked reduction in floral size from the large‐flowered clade (e.g., *T. schlechteri*) to *Papuodendron* species suggests a niche divergence following the radiation of the Papuan group in New Guinea.

Notably, *T*. *borneense* is consistently placed outside *Hibiscus* section *Azanzae* and represents an independent evolutionary lineage within the tribe Hibisceae. Although this species has been considered within *Talipariti* (Fryxell 2001) and *Hibiscus* section *Azanzae* (Borssum Waalkes 1966), it lacks important diagnostic characters such as the stipules leaving annular scars and 10‐celled capsules. These findings highlight the need for a comprehensive taxonomic revision, and an updated checklist of *Talipariti* and its historically associated species is therefore warranted (Vélez‐Esperilla et al., in prep.).

### 4.2 Contrasting Dispersal Histories Within *Talipariti*

A key result of our biogeographical analyses is the strong sensitivity of model fit to the inclusion of widely distributed species. For the full dataset, support for DEC + J was largely driven by the sea hibiscus group, particularly *T. tiliaceum* , with an estimated jump dispersal rate exceeding that of anagenetic range expansion. This pattern is consistent with the superior fit of model M0 for this lineage, and with previous studies documenting rapid range expansion across oceanic barriers (Yamazaki et al. 2023). The inclusion of such widely distributed species increases uncertainty in ancestral area reconstruction and may obscure lineage‐specific biogeographical signals. Therefore, we recommend partitioned analytical approaches when taxa with contrasting dispersal syndromes are present.

Our analyses help resolve the long‐standing question of how the Papuan group colonised Sahul. In contrast with the sea hibiscus group, biogeographic reconstruction strongly favoured a time‐stratified, distance‐dependent dispersal model (M2). Under this framework, the Malesian region is reconstructed as the ancestral area for deep nodes of *Talipariti* phylogeny, and within the genus, a major vicariance event is inferred along Lydekker's Line, separating the intermediate group (*H. glanduliferus* and *T. macrophyllum* ) in Sunda–Wallacea from the Papuan group restricted to Sahul. This spatial pattern is characteristic of the Sunda‐Sahul floristic exchange documented in numerous Malesian plant lineages (Crayn et al. 2015; Joyce et al. 2021; Holzmeyer et al. 2023) and suggests dispersal in the Papuan lineage was constrained by palaeogeographical configuration rather than dominated by stochastic long‐distance dispersal.

Dispersal across Wallacea and its barriers has been documented in numerous plant lineages (Crayn et al. 2015; Joyce et al. 2020). Our results not only add new lineages to this list but also shed light on the nature of these barriers. The better fit of model M2 compared to the strict model M1 demonstrates that Lydekker's Line did not function as an impermeable barrier to dispersal, as traditionally conceived, but rather as a permeable filter whose connectivity varied through time and the dispersal abilities of different lineages. In Malesian lineages without long‐distance dispersal ability as the Papuan group, stepping‐stone range expansion is the dominant dispersal mode, a floristic exchange limited to the Neogene periods of islands emergence and increasing proximity among landmasses.

While our biogeographical analyses provide strong macroevolutionary evidence for palaeogeographically constrained dispersal in the Papuan group, the specific seed dispersal mechanisms underlying this pattern remain to be characterised experimentally. Unlike the sea hibiscus group, where dispersal traits have been empirically verified (Kudoh et al. 2006, 2013), similar studies for the Papuan group are currently limited. This limitation largely reflects the lack of viable fresh material, as seeds from herbarium specimens are desiccated and their original buoyancy and structural properties are often compromised.

Beyond dispersal histories, post‐dispersal establishment represents a critical filter shaping macrobiogeographical outcomes. For instance, in Combretaceae, high dispersal abilities are associated with niche conservatism, restricting sea‐drifted dispersed lineages to coastal and mangrove systems despite wide geographical ranges (Maurin et al. 2023). Similarly, the sea hibiscus group is restricted to coastal environments and exhibits conservative morphology across species while having a pantropical range. In contrast, the Papuan group displays remarkable morphological and ecological diversity, suggesting that in this group, the limited dispersal favoured local adaptation and subsequently niche divergence.

Together, these results demonstrate that differences in dispersal histories among *Talipariti* lineages (as inferred from their contrasting geographical ranges) are associated with fundamentally different macrobiogeographical outcomes across Malesia.

### 4.3 Insights in Biogeographical Model Comparison

Modern biogeographic modelling enables explicit evaluation of competing processes, including dispersal, vicariance, extinction and jump dispersal. This contrasts with earlier frameworks that assumed a priori the predominance of a single process (Matzke 2013a, 2013b). Although the DEC + J framework has been criticised for potentially overemphasising jump dispersal (Ree and Sanmartín 2018), model comparison remains a useful tool for identifying dominant processes when applied in a comparative and lineage‐specific context (Matzke 2022). In the present case, contrasting model support between the sea hibiscus and Papuan groups reflects fundamentally different evolutionary histories rather than a methodological artefact.

The strong sensitivity of model fit to the inclusion of pantropical species is itself informative, and is consistent with the known tendency of DEC + J to favour jump dispersal (Ree and Sanmartín 2018). Under DEC without the jump dispersal parameter, a pantropical distribution spanning areas separated by open ocean would require implausibly high anagenetic dispersal rates (d) across absolute barriers; while DEC + J accommodates such histories by modelling discrete colonisation events at cladogenetic nodes (Matzke 2014).

When the analysis was restricted to the Papuan group, removing the strong signal generated by the pantropical range expansion of *T. tiliaceum* , models without founder‐event speciation were favoured, with DEC and DEC + J returning statistically equivalent likelihoods, indicating that jump dispersal played no significant role.

Nonetheless, model selection based on likelihood methods has limitations for complex datasets; for instance, in our full dataset, having lineages with contrasting dispersal histories. Particularly in clades combining heterogeneous evolutionary processes and dispersal abilities, recent reviews have also highlighted that biogeographic inferences remain strongly dependent on model assumptions, taxon sampling, and the quality of phylogenetic and distributional data (Landis et al. 2026).

### 4.4 Temporal Framework for Malesian Malvoideae Diversification

The extensive fossil record (ca. 80 fossils) has enabled broad temporal calibrations of Malvales (Hernández‐Gutiérrez and Magallón 2019), but fossils with clear affinity to the extant Malvoideae lineage remain scarce. Until recently, *Malvaciphyllum macondicus* represented the only widely accepted calibration point applicable to Malvoideae and has therefore been repeatedly used in divergence‐time analyses (Areces‐Berazain and Ackerman 2016; Hernández‐Gutiérrez and Magallón 2019; Hernández‐Gutiérrez et al. 2022; Yamazaki et al. 2023; Jung et al. 2024).

Here, we incorporate the recently described fossil *Uiher karuen*, characterised by an infructescence with schizocarps diagnostic of the tribe Malveae, providing an additional internal calibration point and improving temporal resolution across major Malvoideae lineages. Our estimated crown age of Malvoideae (66.6 Ma) closely matches previous estimates based on the broader Malvales fossil record (Hernández‐Gutiérrez and Magallón 2019), while being slightly older than estimates based on plastome or reduced‐loci datasets (Areces‐Berazain and Ackerman 2016; Hernández‐Gutiérrez et al. 2022; Yamazaki et al. 2023; Jung et al. 2024). Although these dates rely on plastome data, the strong topological congruence between plastid and nuclear datasets at the deeper nodes (sections, genera and major groups) used for calibration suggests that cytonuclear discordance has not significantly biassed our divergence‐time estimates.

Crucially, our analyses provide the first divergence‐time estimates for the divergence of *Talipariti* (19.7 Ma), and its later diversification originating the Papuan group (6.5 Ma). Also, divergence times are concordant with previous fine‐scale phylogenomic analyses of the sea hibiscus group (Yamazaki et al. 2023). Our estimates establish a temporal framework for evaluating alternative biogeographical scenarios across the Sunda‐Sahul region. These estimates also indicate that major diversification events within *Talipariti* occurred during the Neogene, coinciding with profound tectonic and environmental changes in Malesia.

### 4.5 Phylogenomic Challenges: Gene Tree Discordance and the Utility of Herbariomics

Our study underscores the efficacy of herbariomics for reconstructing evolutionary history in tropical plant groups where obtaining fresh material is difficult. Despite DNA degradation, this approach enabled robust inference of deep phylogenetic relationships and biogeographical history from historical specimens.

At the same time, our analyses confirm that incomplete lineage sorting represents a major source of phylogenetic incongruence in *Hibiscus* section *Azanzae* and *Talipariti*, consistent with broader angiosperm and Malvaceae‐wide patterns (Hernández‐Gutiérrez et al. 2022; Colli‐Silva et al. 2025; Huang et al. 2025). This discordance is most pronounced at shallow nodes, whereas major clade structure and temporal and biogeographical patterns remain robust despite phylogenetic uncertainty. The non‐monophyly of species such as *T. tiliaceum* further underscores the prevalence of ILS in these lineages; however, these taxa may also represent paraphyletic progenitor species from which derived lineages arose through recurrent speciation events (Yamazaki et al. 2023). The status of *T. tiliaceum* as a paraphyletic species requires further investigation combining multiple lines of evidence, including morphological, ecological, and biogeographical data with this phylogenomic framework (Vélez‐Esperilla et al., in prep.).

### 4.6 Future Directions

The framework established here, including the palaeogeographical modelling of Malesia and the comparative evaluation of dispersal models across lineages, provides a foundation for broader investigations of Malesian floristic history. Richer palaeogeographical reconstructions incorporating sea‐level fluctuations, ocean currents, palaeoclimatic conditions, ecological niche modelling, would provide a more detailed context in a biome level (Maurin et al. 2023; Licht et al. 2026). Such advances require, however, careful consistency between palaeogeographical and palaeoclimatic datasets, as well as explicit propagation of uncertainty across phylogenetic, biogeographical and geological sources (Licht et al. 2026; Landis et al. 2026). Additionally, ongoing methodological advances including deep learning tools for model selection across lineages offer promising directions for groups with complex or contrasting dispersal abilities (Gutiérrez de la Peña et al. 2026). The use of these techniques may help resolve the uncertainty in ancestral area estimation at deeper nodes that arises when widely distributed species are included, as observed in our full dataset.

### 4.7 Synthesis: Palaeogeography as a Filter Shaped by Dispersal Histories

In summary, this study provides a comprehensive phylogenomic framework for *Talipariti* and allied species, integrating nuclear and plastid data derived largely from herbarium material. By combining time‐calibrated phylogeny with explicit biogeographical model comparison, we demonstrate that contrasting dispersal histories among closely related lineages are associated with fundamentally different biogeographical outcomes across Malesia.

Quantitative, time‐stratified biogeographical models provide a powerful framework for disentangling dispersal mechanisms across Malesia, particularly when closely related lineages exhibit contrasting dispersal histories. In *Talipariti*, the sea hibiscus group is best explained by infrequent but consequential long‐distance dispersal events, whereas the Papuan group shows a history consistent with range expansion constrained by palaeogeography and island connectivity. These findings highlight that the evolutionary fate of plant lineages is not determined by geological history alone, but by its interaction with lineage‐specific dispersal abilities, revealing Wallace's and Lydekker's Lines as permeable filters rather than absolute barriers. While the deep ancestral nodes of the full phylogeny retain some biogeographical uncertainty, the stark contrast in model support between the two lineages demonstrates that the influence of Sunda–Sahul palaeogeography on plant diversification is not uniform, but depends on lineage‐specific dispersal abilities.

## Author Contributions

**Fernando Vélez‐Esperilla:** conceptualization (lead), data curation (lead), formal analysis (lead), funding acquisition (equal), investigation (lead), methodology (equal), project administration (equal), resources (equal), software (equal), validation (equal), visualization (equal), writing – original draft (lead), writing – review and editing (equal). **Hiroshi Noda:** formal analysis (supporting), investigation (supporting), methodology (supporting), resources (equal), writing – review and editing (equal). **Tadashi Kajita:** resources (equal), writing – review and editing (equal). **Koji Takayama:** conceptualization (supporting), formal analysis (supporting), funding acquisition (lead), investigation (supporting), methodology (supporting), project administration (equal), resources (lead), software (equal), supervision (lead), validation (equal), visualization (equal), writing – original draft (supporting), writing – review and editing (equal).

## Funding

This work was supported by the Kyoto University Department of Graduate Studies (DoGS) Overseas Travel Grant, the Sasakawa Scientific Research Grant 2024‐5027, JSPS KAKENHI Grant Number JP21KK0131, JP23K20303, JP25K02335, and the Eco‐LOGIC‐al Network Project of The Tokyo Metropolitan Government. This work was partially supported by the Asahi Glass Foundation.

## Conflicts of Interest

The authors declare no conflicts of interest.

## Supporting information


**Figure S1:** Heatmaps of gene assembly statistics generated by hybpiper based on Angiosperms353 target‐enrichment data. Rows represent samples and columns correspond to the 353 nuclear loci. (Left) Gene recovery efficiency, showing the percentage of the target sequence length recovered for each locus in each sample. (Right) Paralog detection, displaying the number of gene copies identified for each gene after HYBPIPER paralog investigator.
**Figure S2:** Tanglegram showing the topological conflicts between (left) ASTRAL coalescent species tree based on 267 single‐copy nuclear loci, and (right) plastome CDS data (75 concatenated coding regions). Red branches indicate low support (PP < 0.7 or BS < 70%).
**Figure S3:** Tanglegram showing the topological conflicts between nuclear phylogenies based on 267 single‐copy nuclear loci inferred with different approaches: (left) ASTRAL coalescent species tree, and (right) concatenated supermatrix. Red branches indicate low support (PP < 0.7 or BS < 70%).

**Table S1:1** Herbarium specimens samples information.
**Table S1:2** Silica gel preserved samples information.
**Table S1:3** Sequencing data obtained from public repositories. Left, target enriched nuclear genes obtained from PAFTOL and 1KP. Right, assembled plastome sequences.

**Table S2:1** Species distribution matrix for our 6 biogeografic areas in BioGeoBears analysis. (0) absent, (1) present.
**Table S2:2** Manual dispersal multipliers for three dispersal scenarios (M0‐M2) during different timeslices for BioGeoBears.
**Table S2:3** LnL, AIC and LRT values of the different models used by BioGeoBEARS for the ancestral area estimation in three dataset subsets (Full, sea hibiscus group and papuan group) and three dispersal scenarios (M0–M2).

**Table S3:** MCMC convergence stats.

## Data Availability

Data obtained in this study have been uploaded to Dryad (DOI: 10.5061/dryad.h18932029) and public repositories including NCBI genebank and DDBJ under the same BioProject accesion: PRJNA1496457. Additional information can be directly obtained by contacting the first author.
