# Supplementary figures and images for: Contrasting Dispersal Histories Shape Distinct Evolutionary Trajectories Between Malesian and Pantropical Talipariti (Malvaceae)
Source: Ecol Evol. 2026 Jul 30;16(8):e74056. doi: 10.1002/ece3.74056 (PMC13421916; doi:10.1002/ece3.74056)

FIGURE S1

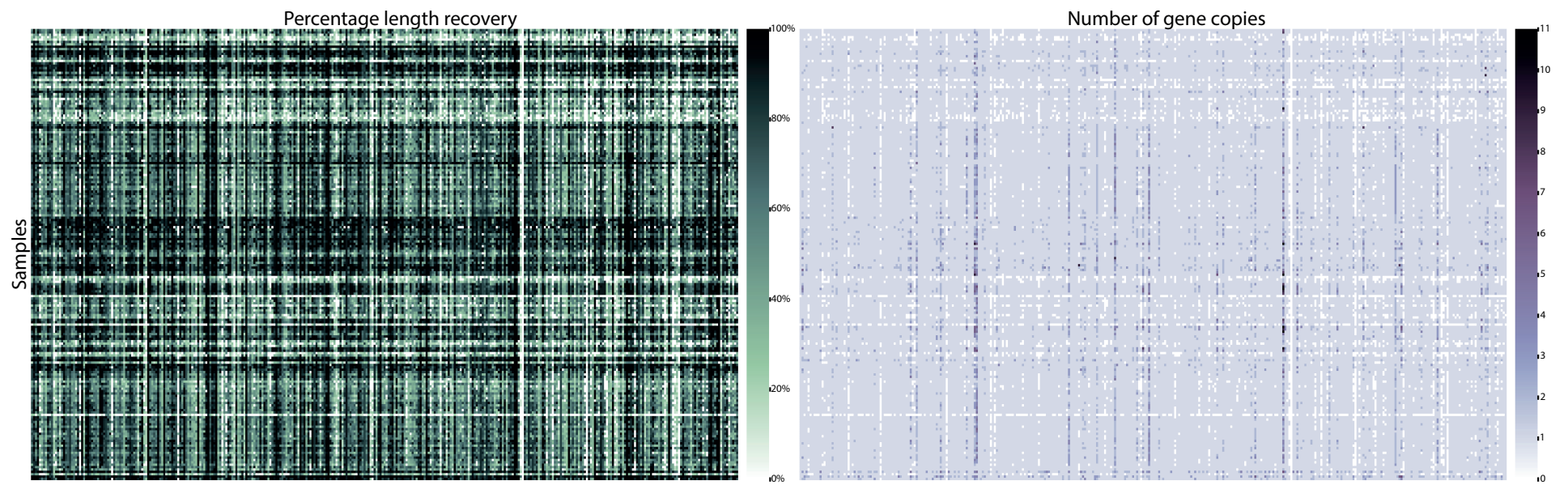

FIGURE S2

## Coalescent nuclear tree

## Concatenated nuclear tree

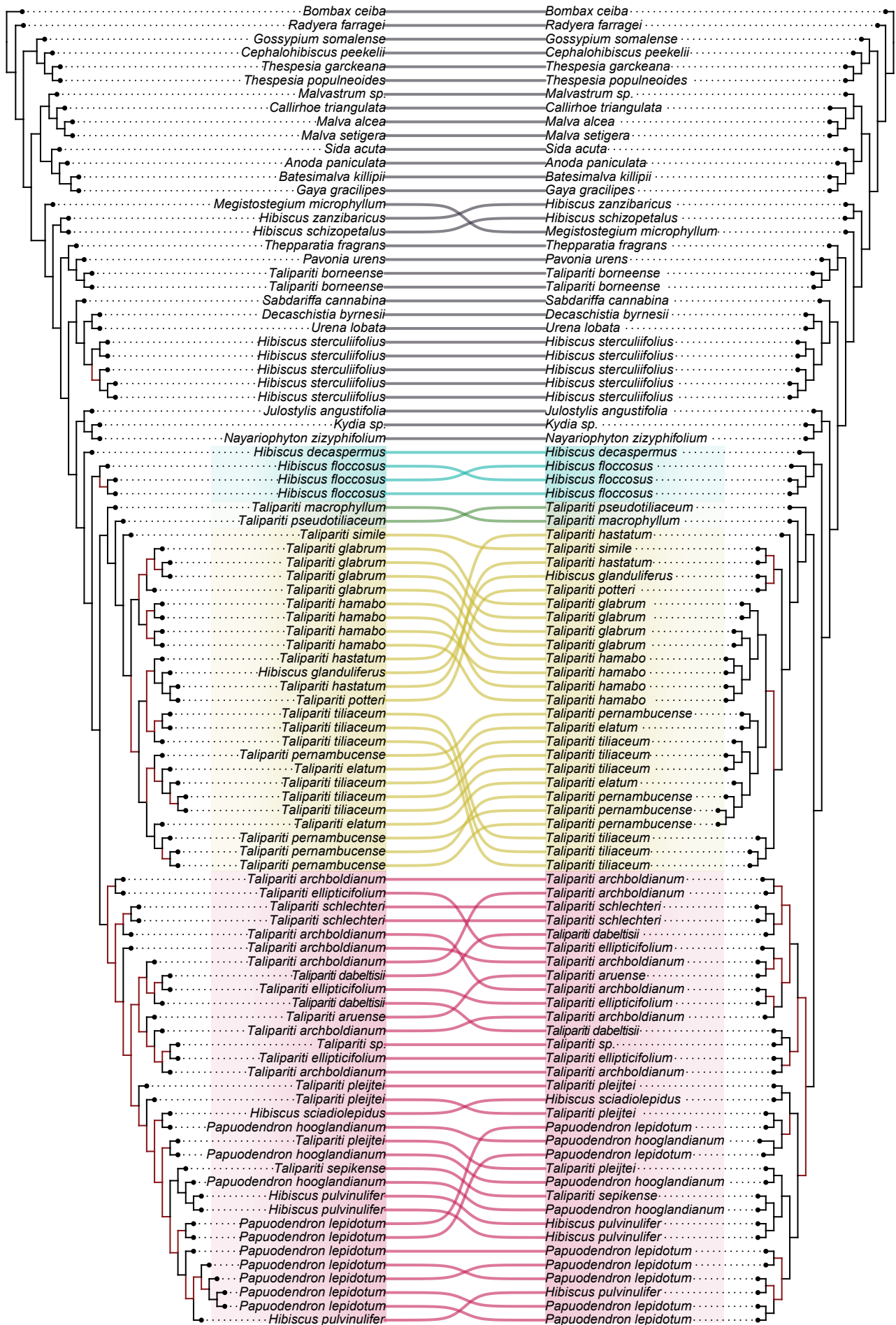

FIGURE S3

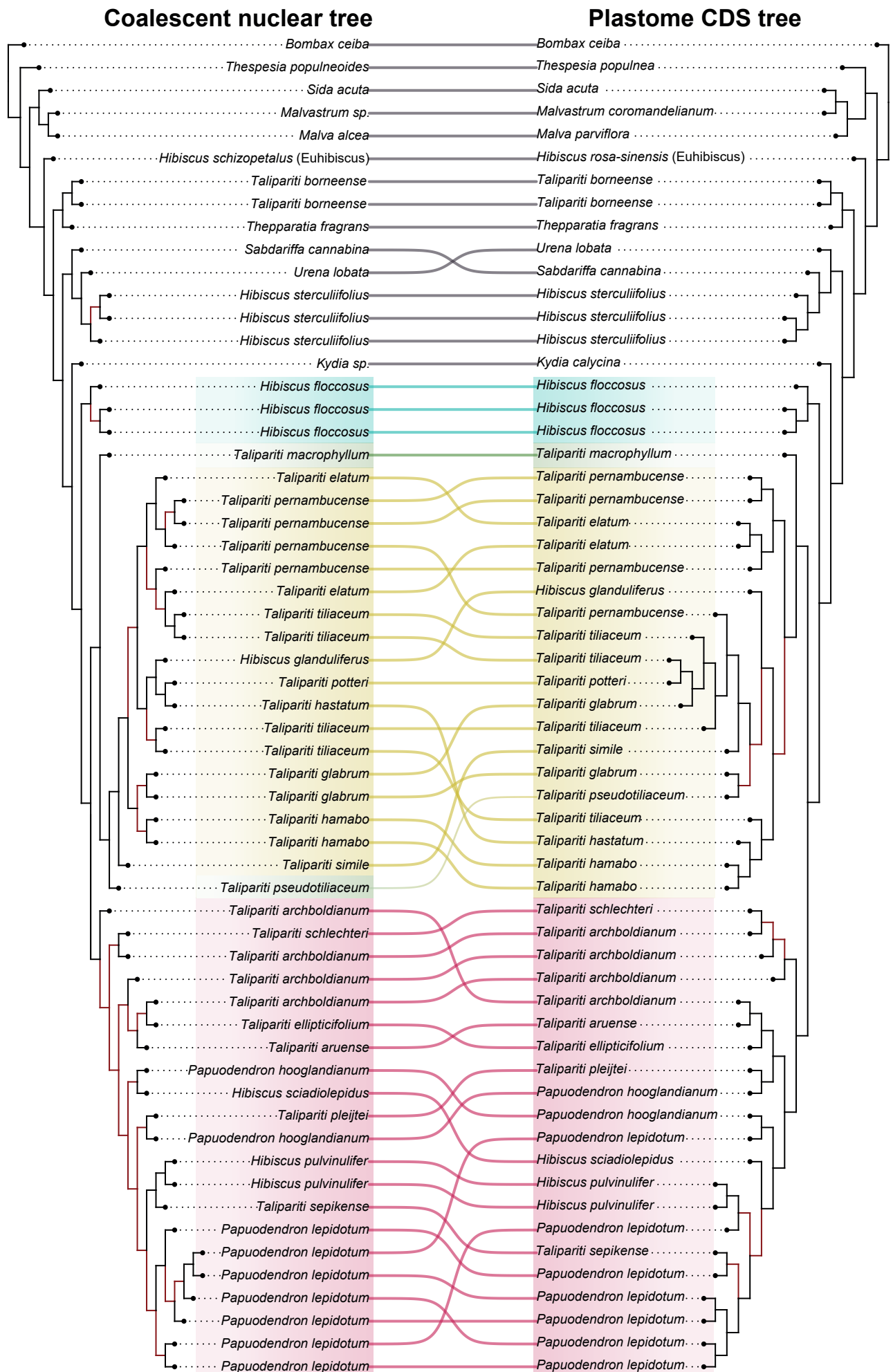

Supplement: Supplementary file 1 — Figure S1: Heatmaps of gene assembly statistics generated by hybpiper based on Angiosperms353 target‐enrichment data. Rows represent samples and columns correspond to the 353 nuclear loci. (Left) Gene recovery efficiency, showing the percentage of the target sequence length recovered for each locus in each sample. (Right) Paralog detection, displaying the number of gene copies identified for each gene after HYBPIPER paralog investigator. Figure S2: Tanglegram showing the topological conflicts between (left) ASTRAL coalescent species tree based on 267 single‐copy nuclear loci, and (right) plastome CDS data (75 concatenated coding regions). Red branches indicate low support (PP < 0.7 or BS < 70%). Figure S3: Tanglegram showing the topological conflicts between nuclear phylogenies based on 267 single‐copy nuclear loci inferred with different approaches: (left) ASTRAL coalescent species tree, and (right) concatenated supermatrix. Red branches indicate low support (PP < 0.7 or BS < 70%). [file ECE3-16-e74056-s003.pdf]
